# Willardiine and Its Synthetic Analogues: Biological Aspects and Implications in Peptide Chemistry of This Nucleobase Amino Acid †

**DOI:** 10.3390/ph15101243

**Published:** 2022-10-10

**Authors:** Rosanna Palumbo, Daniela Omodei, Caterina Vicidomini, Giovanni N. Roviello

**Affiliations:** Institute of Biostructures and Bioimaging-Italian National Council for Research (IBB-CNR), Via De Amicis 95, 80145 Naples, Italy

**Keywords:** willardiine, glutamate receptor, kainate receptor, nucleoamino acid, nucleopeptide, peptide, nucleobase, synthetic compounds, amino acids

## Abstract

Willardiine is a nonprotein amino acid containing uracil, and thus classified as nucleobase amino acid or nucleoamino acid, that together with isowillardiine forms the family of uracilylalanines isolated more than six decades ago in higher plants. Willardiine acts as a partial agonist of ionotropic glutamate receptors and more in particular it agonizes the non-N-methyl-D-aspartate (non-NMDA) receptors of L-glutamate: ie. the α-amino-3-hydroxy-5-methyl-4-isoxazole-propionic acid (AMPA) and kainate receptors. Several analogues and derivatives of willardiine have been synthesised in the laboratory in the last decades and these compounds show different binding affinities for the non-NMDA receptors. More in detail, the willardiine analogues have been employed not only in the investigation of the structure of AMPA and kainate receptors, but also to evaluate the effects of receptor activation in the various brain regions. Remarkably, there are a number of neurological diseases determined by alterations in glutamate signaling, and thus, ligands for AMPA and kainate receptors deserve attention as potential neurodrugs. In fact, similar to willardiine its analogues often act as agonists of AMPA and kainate receptors. A particular importance should be recognized to willardiine and its thymine-based analogue AlaT also in the peptide chemistry field. In fact, besides the naturally-occurring short nucleopeptides isolated from plant sources, there are different examples in which this class of nucleoamino acids was investigated for nucleopeptide development. The applications are various ranging from the realization of nucleopeptide/DNA chimeras for diagnostic applications, and nucleoamino acid derivatization of proteins for facilitating protein-nucleic acid interaction, to nucleopeptide-nucleopeptide molecular recognition for nanotechnological applications. All the above aspects on both chemistry and biotechnological applications of willardine/willardine-analogues and nucleopeptide will be reviewed in this work.

## 1. Introduction

**Figure 1:**
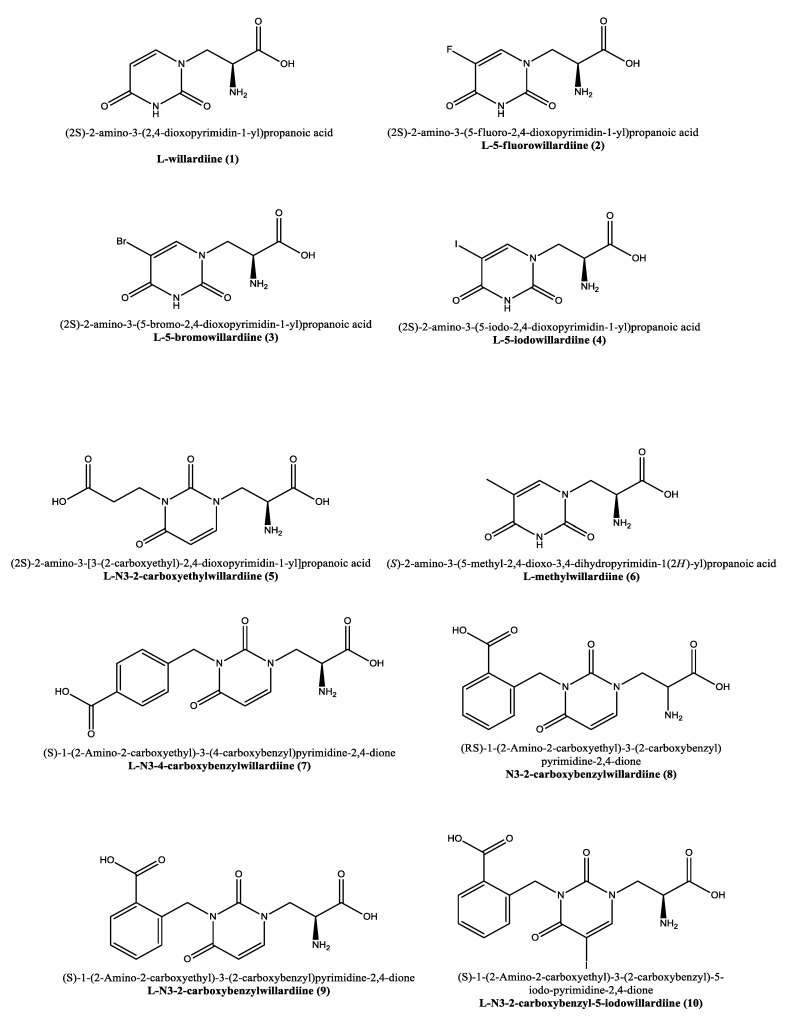
Molecular representation of the structures of willardiine and some of its analogues. IUPAC and use names are reported alongside with the numbering used in this manuscript.

Willardiine was first identified by Rolf Gimelin in 1959 from the extracts of seeds of Acacia willardiana [1]. Structurally it corresponds to (2S)-2-amino-3-(2,4-dioxopyrimidin-1-yl)propanoic acid (**1**, Figure 1) and carrying an uracil moiety it can be ascribed to the category of nucleoamino acids [2,3,4]. Willardiine is synthesized by the single specific enzyme uracilylalanine synthase, and the N–heterocyclic moiety uracil obtained by the orotate pathway [5] proved to be an effective bioisostere for the distal carboxyl group of L-glutamate [6].

This is the main excitatory neurotransmitter found in the central nervous system (CNS) and mediates its actions through a variety of metabotropic (G-protein coupled) and ionotropic (ligand-gated cation channels) receptors [7]. Among the others, ionotropic glutamate receptors are proteins present in almost all mammalian brain structures at the excitatory synapses that facilitate signal transmission in the central nervous system mediating the fast excitatory neurotransmission, and are involved in human nervous system development and function, with their dysfunction being correlated with several CNS disorders [8,9,10,11,12,13]. Mechanistically, antagonists of ionotropic glutamate receptors prevent the closure of the bilobed ligand-binding domain while full agonists close it. In fact, extracellular cleft closure of ionotropic glutamate receptors is associated with receptor activation; however, the mechanism underlying partial agonism is not necessarily linked to the blockade of the full receptor cleft closure; instead, partial agonists more likely destabilize the cleft closure as suggested by in silico studies [14]. There are three types of ionotropic glutamate receptors: the N-methyl-D-aspartate (NMDA) receptor, a glutamate receptor and ion channel present in neurons, and the α-amino-3-hydroxy-5-methyl-4-isoxazole-propionic acid (AMPA) [15] and kainate receptors [16,17,18]. It was demonstrated that in mammalians **1** agonizes non-NMDA glutamate receptors acting as a partial agonist of ionotropic glutamate receptors [19] and in particular activates the AMPA receptors [20,21,22].

Similar to willardiine, its analogues (Figure 1) often act as agonists of AMPA and kainate receptors, but due to their different binding affinities for each receptor, they have been employed not only to investigate the structural and functional consequences of activation of AMPA/kainate receptors, but also to determine the structural modifications required in order to convert **1** into an antagonist at AMPA and kainate receptors [23].

Thus, in this review we describe some of the main ligands, including competitive agonists and antagonists of glutamate receptor family members developed within the willardiine family alongside with the reported structure–activity hypotheses that can serve as starting point for the discovery of new analogues of neurotherapeutic importance with increased selectivity and/or potency properties as binders of kainate or AMPA receptors. Moreover, the role of willardiine and its analogues like the L-methylwillardiine (AlaT) in the peptide chemistry will be discussed in the light of the potential applications of the derived nucleopeptides [24] in diagnostics and biotechnology [25]. 

## 2. Willardiine and Its Analogues

### 2.1. Disease Relevance and Potential Pharmaceutical Role

Willardiine analogues may bind specifically to kainate and AMPA receptors, which are implicated in a variety of neurological disorders characterized by alterations in glutamate signaling. For example, the selective antagonists for glutamate receptor subtypes disclose therapeutic importance for a variety of neurological disorders characterized by aberrant activation of kainate or AMPA receptors. Though far from being exhaustive, in the following section we report on some neurological disorders characterized by dysregulation in either AMPA or kainate receptor activation. 

#### 2.1.1. AMPA Receptors in Neurological Disorders

AMPA receptors are particularly implied in neurodegenerative disorders through their connection with synaptic plasticity, which plays a fundamental role in many physiological aspects of cognitive abilities but also in neural development [26]. Many neuropathies are associated with the cognitive decline, which in turn is linked to changes in AMPA-mediated plasticity [27,28]. In animal models of Parkinson’s disease, enhanced levels of AMPA receptors have been revealed in affected regions, while AMPA antagonists have been proposed as potential therapeutics. However, the benefits of inhibiting AMPA receptors with respect to curing the symptoms of Parkinson’s disease do not always justify the use of this strategy that can cause severe off-target effects [29,30]. On the other hand, AMPA receptor deficits have been correlated with Huntington’s disease, that causes the progressive degeneration of neural cells in the brain [31], as demonstrated in studies conducted in human postmortem tissues and animal models. In particular, modulating AMPA receptors in animal models led to a decrease of degeneration in both the striatum and memory deficits [29]. Moreover, genetic alterations leading to AMPA receptor GluA2 subunit defects were correlated with autism spectrum disorders [32]. The role of AMPA receptors in autism is also supported by the effectiveness of the pharmacological modulation of this type of receptors which rescued social impairments in animal models [33]. Among the many hypotheses about the biological basis of the major depressive disorder, the glutamate hypothesis was corroborated by the evidence that NMDA receptor antagonists blocking the activation of NMDA receptors, and thus causing the AMPA receptor activation to compensate for the decrease in glutamate signaling, have antidepressant effects in animal models [34]. Interestingly, AMPA receptors mediate also the dysregulated sleep, that is a major symptom of major depressive disorder [35]. 

#### 2.1.2. Kainate Receptors in Neurological Disorders

Recent biochemical and behavioral studies suggest a key role for the kainate receptor GluK2 in controlling abnormalities related to the behavioral symptoms of mania, such as psychomotor agitation, aggressiveness and hyperactivity [36], while the kainate receptor GluK3 was associated with recurrent major depressive disorder [37]. Kainate receptors are expressed in regions of spinal cord implied in the transmission of sensory stimulation and pain, and their functional dysregulation was linked to pain [38,39]. Several studies led to a variety of linkages between epilepsy and kainate receptors including different and sometimes contradictory mechanisms that clearly demonstrate the difficulty in studying kainate receptors in this respect [39,40,41]. 

### 2.2. Molecular Insights on Bioactivity of Willardiine analogues

As anticipated, willardiine is a partial agonist of ionotropic glutamate receptors [14] and specifically, agonizes non-NMDA glutamate receptors in particular activating the AMPA receptors [15,16,17]. In analogy to willardiine, its most common analogues are also agonists of kainate/AMPA receptors. The addition of a halogen to the uracil moiety affects the binding affinities and stability of the analogues that are generally more stable than **1** and show higher binding affinity for AMPA receptors. Extensive research has been conducted on the 5-fluorowillardiine (**2**, Figure 1) that showed limited effects at the kainate receptor but acted as a selective agonist of the AMPA receptor and is widely employed in vitro to selectively stimulate AMPA receptors [42,43,44,45,46,47,48]. The activity of the halogen-based analogues **2–4** (Figure 1) at AMPA/kainate receptors was also assayed using mouse embryonic hippocampal neurons [42]. In particular, 5-bromowillardiine (**3**) acted as a potent agonist, and led to rapid but incomplete desensitizing responses. As for the 5-iodowillardiine (**4**) it resulted a selective kainate receptor agonist [49]. On the other hand, **2** with an 1.5 µM EC_50_ was seven times more potent than AMPA (EC_50_ = 11 µM) and 30 times more potent than **1** (EC_50_ = 45 µM); as for the potency sequence within the explored halogen derivatives the observed trend was F(**2**) > Br(**3**) > I(**4**) > willardiine (**1**, Table 1) [42].

Moreover, cross-desensitization experiments revealed that halogen-bearing willardiines bind with different affinity to desensitized receptors that are the same receptors activated by kainate and AMPA, and the rapidly desensitizing and equilibrium responses to the analogues under investigation were mediated by the same receptors. However, they produced different degrees of desensitization with the desensitization sequence being F(**2**) > willardiine (**1**) > Br(**3**) > I(**4**). More in detail, the Iodine-containing derivative (**4**) blocked the activation of the desensitizing response elicited by **1** and **2**, while **1** and **2** blocked the equilibrium response to **4**. It was possible to conclude that simple changes in the molecular structure of willardiine can lead to marked differences in the ability of agonists to produce desensitization of AMPA/kainate receptors [42].

The thermodynamics connected with the interaction of willardiine and its analogues with the ionotropic receptors was studied in order to obtain useful insights exploitable for new drug design [38]. For example, analogues of willardiine modified at position 5 with F, Cl, I, H and NO_2_ led to deeper insights on the thermodynamics of the interaction of willardiine with AMPA receptors [50]. The binding of willardiine analogues to subtypes of the AMPA receptor was, in some cases, driven by increases in entropy. The major part of the studied analogues were partial agonists whose charged state was found in direct connection with the enthalpic contribution to the interaction [38]. In particular, the binding of the charged forms to AMPA receptor is driven essentially by enthalpy, while the interaction of the uncharged form is largely dominated by the entropic contribution due to changes in the hydrogen-bonding network within the binding site, with these findings providing clues for further neurodrug development [38].

**Figure 2:**
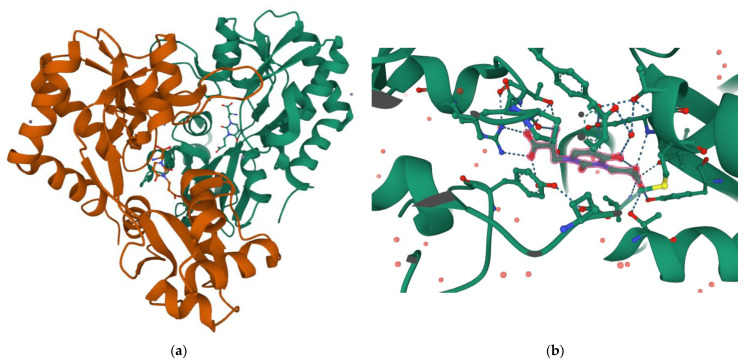
(**a**) 3D view of the crystal structure of the complex between the binding domain of the AMPA subunit GluR2 and **5** (PDB DOI: 10.2210/pdb3H03/pdb PDB ID: 3H03 https://www.rcsb.org/3d-sequence/3H03?assemblyId=1, accessed on 29 August 2022); (**b**) Detailed view of the ligand interaction in the same complex (for more details on protein residues involved in the binding see the link https://www.rcsb.org/3d-view/3H03?preset=ligandInteraction&label_asym_id=E, accessed on 29 August 2022). Note how the amino acid COOH forms a double H-bond with Arg93, while the ethyl COOH forms H-bonds with Tyr187 and Thr171.

Interestingly, the carboxyethyl derivative of willardiine (**5**) was found to act as antagonist of the AMPA receptor and more in detail, the crystal structure of the GluR2 binding domain of this receptor, which is crucial for mediating its calcium permeability, in complex with **5** was described in the literature (Figure 2a,b) [51].

This compound binds to one lobe of the protein with interactions similar to agonists, while the binding with the second lobe produces a stable lobe orientation that is similar to the apo state. The carboxyethyl substituent in the N(3) position of **5** keeps the protein lobes separated and the internal dynamics are minimal compared to the protein bound to the reference antagonist 6-cyano-7-nitroquinoxaline-2,3-dione, which minimizes the contacts with one of the two lobes. This latter complex is less stable than that observed with **5** despite a 100-fold higher affinity [45]. Overall, the study with **5** suggests that the antagonism of willardiine analogues is associated with the overall orientation of the lobes of the AMPA receptor rather than with specific interactions [45]. Another study [23] conducted on a range of novel willardiine analogues as antagonists acting at AMPA and kainate receptors suggested that for a derivative to act as AMPA/kainate receptor antagonist, an N3-substituent bearing a carboxylic acid (like in **5**) side-chain is needed, the S-stereochemistry should be present in the derivative and a iodine added to the 5-position of the uracil moiety enhances antagonism at kainate receptors. Moreover, in the same study the 3-methyl analogue **6** (Figure 1), was found to be a weak agonist, indicating that merely blocking ionisation of the uracil ring of willardiine even though decreases the agonist activity is not sufficient to convert the analogue into an antagonist [23]. Aiming at developing new antagonists of kainate/AMPA receptors also N3-carboxybenzyl substituted willardiine analogues were synthesized [49]. The N3-4-carboxybenzyl derivative (**7**) proved to be equipotent at AMPA and kainate receptors in the rat spinal cord. The racemic N3-2-carboxybenzyl analogue (**8**) was found to be a potent and selective kainate receptor antagonist in experiments conducted on native rat and human recombinant kainate and AMPA receptors. More in detail, the kainate receptor antagonist activity was demonstrated to reside essentially in the S enantiomer (**9**). 5-Iodo substitution of the uracil ring of **9** gave **10**, which proved to have enhanced selectivity and potency for the kainate receptor [49].

## 3. Willardiine in Peptide Structures

In general, synthetic nucleobase-containing molecules [52,53,54,55,56,57,58,59] as well as modified nucleosides [60,61,62,63,64,65,66] are interesting compounds with several biomedical properties [67,68,69]. Introducing single nucleobase-bearing amino acids into polyamino acid chains led to nucleopeptides able to stabilize certain protein and peptide structures, enhancing their function [70,71,72]. These DNA analogues are known not only for their ability to bind complementary nucleic acids [55,57,73] and permeate through cell membranes [55,56,74,75,76,77] but also to self-assemble [78,79], and are endowed with other biomedically-relevant properties [72,80,81]. In this regard, nucleoamino acids [56], made up of amino acids carrying DNA or RNA bases, are useful building blocks of modified peptides with improved biomolecular binding properties. Examples of natural nucleoamino acids are given not only by **1**, but also lathyrine, a non-proteinogenic amino acid from *Lathyrus* species, and discadenine, a plant cytokinin isolated from *Dictyostelium discoideum*. Refs. [82,83,84,85] Nonetheless, nucleoamino acid moieties are found in the natural peptidyl nucleosides [86], molecules that play a key role in biology and therapy, while a plethora of synthetic nucleoamino acids were developed as building blocks of nucleopeptides [87,88,89,90,91,92].

**Figure 3:**
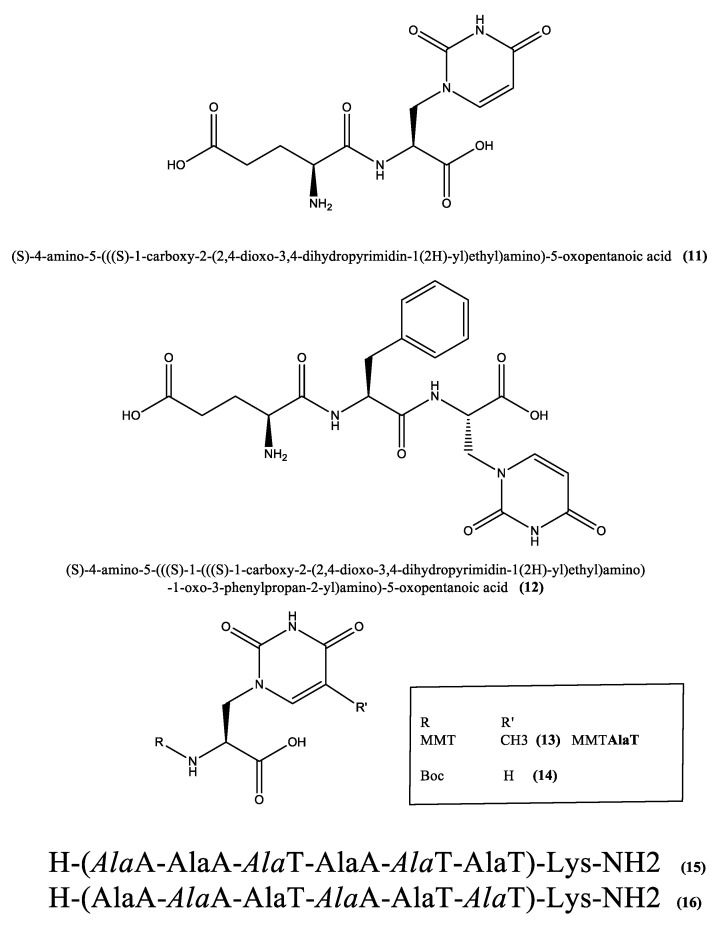
Examples of peptides containing willardiine and the derivative **6** herein referred to as AlaT, whose structure as N-protected monomer for solid phase oligomerization is also reported (**13**, **14**). A and T stand for adenine and thymine nucleobases, respectively. Italics refer to D-configuration of the amino acid residues in the sequences **15** and **16**.

Examples of natural nucleopeptides containing **1** in their structures are given by γ-glutamylwillardiine **11** and γ-glutamylphenylalanylwillardiine **12** (Figure 3) that were isolated from vegetal sources and whose structures were established by hydrolytic procedures to give the constituent amino acids, and for the tripeptide, the constituent dipeptides, and spectroscopy [93]. The compound **1** in its Nα-acetyl-modified form was obtained synthetically as a racemic mixture and subsequently deacylated enzymatically to the free L-willardiine by treatment with an acylase enzyme [94]. The molar ellipticity at 260 nm (−2.0 M^−1^cm^−1^) was in agreement with the expected value for the pure enantiomer. After these steps, several peptides containing **1** were synthesized through solid phase peptide strategy using an oxim resin. In particular, they were obtained alternating **1** and glycine and placing a dansyl fluorophore at the N-terminus as a fluorescence probe. Remarkably, the willardine-based peptides showed β-sheet structures in aqueous solution as revealed by CD analysis [94].

The utilization of analogues of **1** in building up enzyme-resistant peptides in which specific amino acid residues of the native sequence were replaced by β-nucleo-α-alanines was reported in the literature [46]. Research efforts focused on this type of studies were devoted especially by the group led by prof. Ulf Diederichsen of University of Goettingen in Germany. They introduced the analogues of **1** into modified ανβ3-integrin-inhibiting RGD cyclopeptides, combining, thus, the properties of the peptide backbone with the properties inherent the heterocyclic nucleobases [2]. They also designed alanyl-nucleopeptide/DNA chimeras for DNA diagnostics whose solid phase synthesis protocol required the introduction of nucleotides as phosphoramidites, while the nucleoamino acids as monomers protected by the acid labile monomethoxytrityl (MMT) group on the α-amino groups, and by acyl protecting groups on the exocyclic amino nucleobase groups. In this context, they synthesized the four MMT/acyl-protected nucleo alanines, including the MMT-protected willardiine analogue AlaT (**13**, Figure 3), that are useful monomers for the obtainment of nucleopeptide/DNA chimeras under conditions that are compatible with the standard phosphoramidite DNA synthesis strategy [95]. Interestingly, nucleopeptides containing analogues of **1** such as AlaT, are endowed with cell-membrane permeability and were also able to reach the cell nucleus without exerting any cytotoxic effects [75].

Since proteins which bind nucleic acids, employing different strategies for the recognition of the structural elements in their nucleic acid targets, are able to regulate their structure and functions, their preparation with nucleoamino acid incorporations into the protein structure, aiming at reinforcing their nucleic acid-binding ability, was described in the literature [96]. The introduction of nucleoamino acid units with strategies using the Boc-protected AlaT **14** (Figure 3) into the structures of dihydrofolate reductase and the Klenow fragment of DNA polymerase I was accomplished with acceptable efficiencies [96]. In another work, nucleopeptide/nucleopeptide pairing properties and different stacking modes were studied using peptides containing AlaT moieties like those corresponding to the sequences **15** and **16** (Figure 3) [97]. Overall, the different types of nucleopeptides studied, including both L- and D-nucleoamino acid residues, proved to be valuable model systems for the investigation of processes based on nucleobase-stacking like interactions with the base stack, intercalation and electron transfer, and the findings of this study indicated that the extension of a side chain linker by a methylene group in the L-willardiine structure does affect the functional unit regarding facial orientation and also the order of donor/acceptor positions [97]. AlaT units were used also in the synthesis of short, β-turn- or helix-forming, nucleopeptides containing 2-aminoisobutyric acid (Aib). Ref. [98] More in detail, AlaT units were introduced into homo-peptide stretches. In this regard, an hexamer sequence was designed and realized in order to allow the alignment of two DNA bases on the same face of the helical structure [98]. The most interesting features of these AlaT-containing nucleopeptides emerged from this research work included a particularly rigid peptide backbone that formed helices or β-turn structures, in dependence of the main-chain length, a backbone-to-side chain H-bond limiting the nucleobase mobility and a high tendency to form dimers that were mediated by the nucleobases [98]. The synthesis of the AlaT monomer for the peptide oligomerization was accomplished starting from the L-serine β-lactone, while the peptide was synthesised in solution due to the scarce propensity of Aib to form peptide bonds, especially in the case of two or more residues of this hindered amino acid consecutively present in the peptide sequence. NMR studied performed in CDCl_3_ on this type of nucleopeptides revealed their tendency to adopt folded conformations [98]. By introducing two AlaT nucleoamino acids and 0, 1 or 4 Aib units in alanyl-nucleoheptapeptides, and studying the corresponding conformational properties [99], it was possible to conclude that a single Aib amino acid in the sequence is sufficient to promote the adoption of a helical structure in the AlaT nucleopeptides and is enough to markedly reduce their vulnerability towards enzymatic hydrolysis as ascertained in assays conducted in murine serum [99]. On the other hand, introducing four Aib residues, out of seven residues in the nucleopeptide sequence, led to a rigid helical alanyl-nucleopeptide that was almost untouched by serum enzymes and did not show any appreciable cytotoxicity. The reported behaviour probably depends on the fact that the hydrolytic enzymes are more efficient in cleaving peptide bonds among proteinogenic amino acids, rather than amide bonds involving non-coded residues [99]. AlaT was one of the nucleobase-containing residues inserted into the peptide sequences described in a recent work aiming at establishing efficient methods for the chemical synthesis and de novo sequencing of nucleopeptides able to recognize oligonucleotide targets with high affinity [92]. Specifically, combinatorial libraries with up to 100 million biohybrid compounds were prepared and tested against RNA targets, demonstrating that the biohybrid materials bearing nucleobase moieties were endowed with higher bulk affinities for the oligonucleotide target than peptides based exclusively on canonical amino acids. By affinity selection mass spectrometry, it was also possible to discover particular nucleopeptide variants with a high affinity for the specific oligonucleotide targets, that in this studies corresponded to pre-microRNA hairpins [92].

The effect of double strand formation in alanyl-nucleopeptide/protein chimeras on the induction of a conformational switch in proteins and peptides was explored using peptides containing the guanine and cytosine analogues of **1** [100]. The importance of this study stems from the fact that the biological properties of proteins are correlated with their conformation and the three-dimensional arrangement of the secondary structure elements present in them. Moreover, protein-protein binding and conformational changes, such as those caused by molecular switches, may determine consequences in terms of biological function of the protein. Specifically, one type of molecular switches that was inserted in the peptide sequences of this work corresponded to the alanyl-nucleopeptides based on analogues of **1**, and more specifically obtained by the oligomerization of alanine units bearing the guanine and cytosine nucleobases attached to the β-position of the amino acid residues. In this context, the study of several complementary nucleopeptides with different mismatches, residue numbers, and under various buffer conditions was performed by using NMR and UV spectroscopy in order to investigate their interaction abilities and consequently the stability of their complexes [100]. Moreover, willardiine analogues were incorporated in the sequences of β-sheet-forming peptides. From a synthetic perspective, the same work aimed at developing N-methylated nucleoamino acids to be incorporated in the nucleopeptides, by using a convenient route for the synthesis of these N-methylated derivatives starting from N-methyl-serinelactone [100], while the main aim from a biomolecular and applicative viewpoint was replacing the C-terminal helix structure of humane interleukine 8 (IL-8) (residues 56–77) with a nucleopeptide moiety in order to switch from a helical-structured C terminus to another secondary structure determined by the interactions of the alanyl-nucleopeptides based on **1** analogues. However, due to the difficulties found in the incorporation of the nucleoamino acids in the peptides and proteins, probably because of the effects of the nucleobases, an enzymatic ligation was ultimately chosen for the alanyl-nucleopeptide/protein bioconjugation [100]. As for the stability of the nucleopeptide/nucleopeptide duplexes, it was evaluated by varying the number of residues, as well as the sequences of the nucleopeptides. Moreover, AlaT was introduced with the aim of avoiding aggregation problems, together with L- lysine and L-glutamine as mismatch amino acids, that are notoriously able to increase the solubility of the modified nucleopeptides. Subsequently, structural insights on the nucleopeptide constructs, as well as the exploration of their conformational changes were achieved by temperature-dependent NMR and UV spectroscopy-based experiments, finding that a single mismatch was enough to improve or decrease the self-pairing tendency. As expected, a higher aggregation propensity was revealed in pure G/C-nucleopeptides than in the modified oligomers as shown by NMR spectroscopy. In addition, single or multiple insertions of N-methylated nucleoamino acid residues in the center of the alanyl-nucleopeptide sequences led to structures with lower aggregation tendencies [100], while charged N-alkyl chains were introduced in the complementary nucleopeptides to enable NMR-spectroscopy monitoring, blocking the aggregation of the molecules [100]. In order to investigate the effects of nucleopeptide oligomers as molecular switches and in consideration of the fact that two complementary nucleopeptide sequences are able to form a stable double strand with linear topology endowed with a β-strand conformation thanks to the specific complementary DNA base recognition [100], an alanyl-nucleopeptide segment with six residues was incorporated in both a peptide and a protein in order to stabilize a β-sheet conformation in the resulting conjugates. Once inserted in the peptide and protein structures the alanyl-nucleopeptides led to the formation of a double helix that has proven to be a switchable stabilizing or destabilizing element of the resulting peptide or protein structure. Interestingly, when incorporated in β-sheet forming peptides, as well as in proteins, nucleopeptides led to a portion of a β-sheet, as observed in the case of nucleopeptide double strands, or a disturbed β-sheet, as revealed in the case of nucleopeptide single strand [100]. An N-acetylated peptide/nucleopeptide conjugate was firstly synthesised and used in binding experiments with a complementary nucleopeptide, but the expected pairing was not revealed probably because it was prevented by both sterical and electronical factors that involved both the nucleoamino acids and the amino acid residues present in the peptide/nucleopeptide conjugate. With this in mind, a shorter and more flexible peptide that carried a less rigid β-turn element was conjugated to the alanyl-nucleopeptide, and this made it possible to observe a clear nucleopeptide/nucleopeptide pairing in the binding experiment involving the new conjugate and the complementary nucleopeptide as hypothesised [100]. Furthermore, the substitution of the the C-terminal helix part of IL-8 with a peptide sequence that contained an alanyl-nucleopeptide moiety was achieved with the aim to switch from a structured C terminus to another stable secondary structure determined by the nucleopeptide molecular interactions. More in detail, the fragment of human IL-8 (residues 1–55) was expressed in *E. coli* by the ‘Intein Mediated Purification with an Affinity Chitin-binding Tag’ (IMPACT) purification system. Subsequently, a first approach using a native chemical ligation was employed leading to a protein/nucleopeptide conjugate that allowed to switch the protein conformation and to modify the protein binding properties by the nucleobase interactions provided by the alanyl-nucleopeptide moiety [100]. A second approach used an enzymatic ligation for the protein/nucleopeptide conjugation. To this scope, it was necessary to synthesize alanyl-nucleopeptides with different number of residues and N-terminal nucleoamino acid units [100]. The ligation step was perfomed on different sequences using the protease clostripain (from *Clostridium histolyticum*) as enzyme. Overall, the enzymatic ligation proved to be successful in the incorporation of the alanyl-nucleopeptides into the desired protein or peptide [100]. 

To understand the complex relationship between the various interactions involving the nucleic acid pairing nucleobases on which the functions of DNA and RNA mainly depend, model systems are needed in which the interstrand pairing is less restricted for effect of the backbone than in natural oligonucleotides. Examples of systems of this type were offered by alanyl-nucleopeptides whose peptide backbone replaced the sugar phosphate repeats of RNA or DNA. In this context, the group led by prof. Diederichsen devoted enormous efforts to the synthesis of a large number of such model systems based on an alanine-containing backbone to which both canonical and non-canonical bases were anchored and whose monomers, thus, can be seen as willardiine analogues [101]. These nucleopeptide systems were found to aggregate into structures that showed several binding motifs never observed in the case of RNA or DNA. In particular, Diederichsen et al. aimed at studying these uncommon binding motifs to get an insight into the interplay of the interactions between the DNA bases. However, the solubility of the alanyl-nucleopeptide sequences was often too low making it difficult to experimentally elucidate the geometrical arrangements with techniques like NMR or X-ray, and on the other hand UV spectroscopy could provide information solely on the overall stability of the various aggregates through measurements of melting temperatures [101]. Therefore, in the attempt to corroborate the knowledge about the geometrical structure and the bonding motifs of nucleobases and to study the effects which governed the trends in the stabilities of the alanyl-nucleopeptide systems, new insights on the interplay of their various interactions were obtained by theoretical approaches [101]. Specifically, as a simplification this was achieved without evaluating the absolute stabilities, even because many contributions such as dynamic and entropic effects were very similar for close systems and thus, were considered less important in the described approach [101]. Since the noncanonical nucleobases were not implemented in the standard version of the Amber4.1 force field, for the investigation of all experimentally tested willardiine analogues-based nucleopeptides, it was essential to parameterize them, which was performed by adding the missing parameters to the Amber Force Field. The construction of all possible pairing modes for the nucleopeptide model dimer was accomplished at the beginning of the theoretical investigation. However, not all the pairing modes were realizable because of the restriction of the nucleopeptide backbone, as well as the geometrical arrangement of the dimer. For some pairing modes it was possible to obtain a construction, but the geometrical restrictions of the nucleopeptide backbone led to a particularly high strain in the system that made them fall apart in a first geometry optimization step. On the other hand, molecular dynamics (MD) simulations were run on those systems that revealed to be stable [101]. By starting several geometry optimizations at different points of the MD simulation it was possible to get useful information about their geometrical arrangements at zero temperature (T = 0 K) and, in particular, the obtained geometries resulted identical. Moreover, by using a two-steps method in which the influence of the backbone and the effects linked to the DNA bases were evaluated separately, it was possible to describe the interactions that took place within a nucleopeptide dimer at T = 0 K [101]. Overall, the described theoretical study led to an efficient protocol that worked for the description of the nucleopeptides based on willardiine analogues, especially in consideration of the good correlation found between the computed stabilization energies and the measured melting temperature (T_m_) values. In particular, a very good correlation between the experimental T_m_s and the calculated stabilization energies was observed for the respective most stable pairing modes of the dimer formed by the various nucleopeptide oligomers. This suggested that the T = 0 K model depicts accurately the main effects which led to the trends observed in the investigated systems and, thus, that the enthalpic and entropic effects were not important for the relative stabilities of the alanyl-nucleopeptide dimer but only in the case of their absolute stabilities [101].

### 3.1. Charge Transfer and Transport in Nucleopeptides

The research field that studies the charge transfer and transport in nucleic acids occupies an important role in the design of DNA-based devices for molecular electronics, as well as in the investigation of the oxidative damage to double helical oligonucleotides [102]. Interestingly, a study [103] was conducted about the femtosecond time resolved electron transfer dynamics of double-stranded alanyl-nucleopeptides in which both peptide strands contained willardiine analogues and hosted a molecule of 9-amino-6-chloro-2-methoxy-acridine in its protonated state intercalated in the interior of the double strand. In this work several alanyl-nucleopeptides were designed and synthesised in order to realize rigid and linear pairing complexes [103]. From a structural viewpoint, the main results of this study were in agreement with the notion of alanyl-nucleopeptide stacking distances in the base staple similar to the one in the B-DNA model, thus leading to structural evidences for base stacking in double-stranded alanyl-nucleopeptides [103].

### 3.2. Prebiotic Role of Nucleopeptides

The scientific research on the origins of life is a very attractive field and in this context a great relevance is given to prebiotic chemistry according to which some of the chemical precursors of proteins and nucleic acids might be produced under prebiotic conditions [104,105]. Remarkably, nucleopeptides were proposed as potential primordial nucleic material in a scenario preceding the so-called ‘RNA world’, due to their characteristics of self-replicating molecules. On the other hand, the interaction between RNAs and nucleopeptides could have played a fundamental role in the transition from the hypothesised nucleopeptide-driven world to the current DNA, RNA and protein-based genetic system [106]. In this regard, chimeric nucleobase-peptide derivatives were used as models of nucleopeptides to be studied to prove their ability to replicate non-enzymatically, which is a pivotal mechanism on which chemical evolution is based [106]. More in particular, it was shown that the replication of complementary nucleobase-bearing peptides followed various mechanisms with a net selection of a single structure over the several other possible ones sustaining, thus, the hypothesis that similar processes might be at the origin of the first functional nucleopeptide assemblies, which subsequently determined the occurrence of biological structures like ribosomes [106]. Moreover, the processes of selection and self-organization were demonstrated to occur in mixtures of complementary nucleopeptides consisting of a limited number of residues, and specifically in nucleopeptides build-up of eight amino acids conjugated at their N-termini to thymine and adenine bases through carboxymethylene bridges. Both theoretical simulations and experimental studies were conducted on these molecules, with their main results suggesting that the template-directed replication processes of cross-catalytic and autocatalytic type took place within these networks leading to the formation of the products [106]. 

Alanyl-nucleopeptides based on willardiine might have been involved in a prebiotic scenario. In fact, this nucleoamino acid is not only a metabolic product of plants but also a potential prebiotic product. In particular the prebiotic synthesis of willardiine was demonstrated to occur via pyridoxal-5′-phosphate (PLP)-catalyzed derivatization of O-acyl L-serine or L-serine with the appropriate base that in case of willardiine is uracil [107]. Notwithstanding the hypothesised ability of willardiine-based nucleopeptides with an appropriate arrangement of the bases to recognize a complementary sequence of the natural nucleic acid, which is considered a requirement in the transition from the prebiotic nucleopeptide world to the current based on DNA, RNA and proteins, the nucleopeptides synthesized initially to this scope failed to recognise effectively their complementary oligonucleotide strands, probably due to the use of racemic monomers. Nonetheless, several synthetic efforts including those aimed at controlling the chirality of the backbone led to nucleopeptides that could bind the complementary natural nucleic strands [107].

## 4. Conclusions

Following the enormous efforts devoted to modifying the structure of uracylalanines to obtain new glutamate receptor binders, a large number of willardiine analogues is now available. Some of them act as agonists of ionotropic glutamate receptors agonizing especially the non-NMDA glutamate receptors. They showed different binding affinities for the non-NMDA receptors and had a key role in the structural studies of AMPA and kainate receptors, but also in the investigation of the specific effects caused by the receptor activation in the different brain regions. Many neurological disorders are determined by alterations in glutamate signaling, and thus, ligands for AMPA and kainate receptors like the willardiine analogues deserve attention as potential therapeutics. Some halogen-based willardiine analogues show binding affinities and stability significantly higher than the natural willardiine, as emerged in studies with the AMPA receptor. On the other side, synthetic carboxylbenzyl-substituted willardiine analogues have proved to be AMPA and kainate glutamate receptor antagonists, with therapeutic potential for a variety of neurological diseases caused by the aberrant activation of kainate or AMPA receptors.

Not less importantly, the willardiine and its thymine-based analogue AlaT have a key role in peptide chemistry. In fact, besides the natural γ-glutamylwillardiine and γ-glutamylphenylalanylwillardiine, isolated from vegetal sources, there are different examples in which the nucleoamino acid and its analogue AlaT were employed for nucleopeptide development. The applications are various including nucleopeptide/DNA chimera fabrication for DNA diagnostics, nucleoamino acid-derivatization of proteins for facilitating protein-nucleic acid interaction, and nucleopeptide-nucleopeptide interaction as model systems in various structural studies or for nanotechnological strategies. All the above aspects on chemistry and biotechnological applications of both willardine analogues and the corresponding nucleopeptides encourage clearly future efforts for the development of novel willardiine-inspired drugs able to interact with receptor family members in CNS function with greater selectivity for individual receptor subunits and as potentially novel therapeutics.

## Figures and Tables

**Table 1:** Steady-state EC_50_ ^1^ for AMPA/kainate receptor activation by willardiines.

Compound	EC_50_ (μM)	±SD
**1**	44.8	15.0
**2**	1.47	0.39
**3**	8.82	1.29
**4**	19.2	1.92

^1^ adapted from data reported in [42].

## Data Availability

Data sharing not applicable.
